# Asia expert consensus on segmentectomy in non–small cell lung cancer: A modified Delphi study

**DOI:** 10.1016/j.xjon.2023.03.013

**Published:** 2023-04-07

**Authors:** Lunxu Liu, Keiju Aokage, Chang Chen, Chun Chen, Liang Chen, Yong-Hee Kim, Chang Young Lee, Chengwu Liu, Chia-Chuan Liu, Wataru Nishio, Kenji Suzuki, Lijie Tan, Yau-Lin Tseng, Masaya Yotsukura, Shun-ichi Watanabe

**Affiliations:** a Department of Thoracic Surgery, West China Hospital of Sichuan University, Chengdu, Sichuan, China; b Division of Thoracic Surgery, National Cancer Centre Hospital East, Chiba, Japan; c Division of Thoracic Surgery, Shanghai Pulmonary Hospital Tongji University, Shanghai, China; d Department of Thoracic Surgery, Fujian Medical University Union Hospital, Fuzhou, China; e Department of Thoracic Surgery, The First Affiliated Hospital of Nanjing Medical University, Jiangsu Province Hospital, Nanjing, China; f Division of Thoracic Surgery, Department of Thoracic and Cardiovascular Surgery, Asan Medical Center, University of Ulsan College of Medicine, Seoul, South Korea; g Department of Thoracic and Cardiovascular Surgery, Severance Hospital, Yonsei University College of Medicine, Seoul, South Korea; h Division of Thoracic Surgery, KOO Foundation Cancer Centre, Taipei, Taiwan; i Department of Chest Surgery, Hyogo Cancer Center, Akashi, Japan; j Department of General Thoracic Surgery, Juntendo University, Tokyo, Japan; k Division of Thoracic Surgery, Zhongshan Hospital, Fudan University, Shanghai, China; l Division of Thoracic Surgery, National Cheng Kung University Hospital, Tainan, Taiwan; m Department of Thoracic Surgery, National Cancer Centre Central Hospital, Tokyo, Japan

**Keywords:** Asia, Delphi, expert consensus, lobectomy, non–small cell lung cancer, segmentectomy, sublobar resection

## Abstract

**Objective:**

Segmentectomy as a parenchymal-sparing surgical approach has been recommended over lobectomy in select patients with early-stage non–small cell lung cancer. This study aimed to address 3 aspects of segmentectomy (“patient indication”; “segmentectomy approaches”; “lymph node assessment”) where there is limited clinical guidance.

**Methods:**

A modified Delphi approach comprising 3 anonymous surveys and 2 expert discussions was used to establish consensus on the aforementioned topics among 15 thoracic surgeons (2 Steering Committee; 2 Task Force; 11 Voting Experts) from Asia who have extensive segmentectomy experience. Statements were developed by the Steering Committee and Task Force based on their clinical experience, published literature (rounds 1-3), and comments received from Voting Experts through surveys (rounds 2-3). Voting Experts indicated their agreement with each statement on a 5-point Likert scale. Consensus was defined as ≥70% of Voting Experts selecting either “Agree”/“Strongly Agree” or “Disagree”/“Strongly Disagree.”

**Results:**

Consensus from the 11 Voting Experts was reached on 36 statements (11 “patient indication” statements; 19 “segmentation approaches” statements; 6 “lymph node assessment” statements). In rounds 1, 2, and 3, consensus was reached on 48%, 81%, and 100% of drafted statements, respectively.

**Conclusions:**

A recent phase 3 trial reported significantly improved 5-year overall survival rates for segmentectomy compared with lobectomy, proposing thoracic surgeons to consider segmentectomy as a surgical option in suitable patients. This consensus serves as a guidance to thoracic surgeons considering segmentectomy in patients with early non–small cell lung cancer, outlining key principles that surgeons should consider in surgical decision-making.

## Graphical abstract

**Figure:**
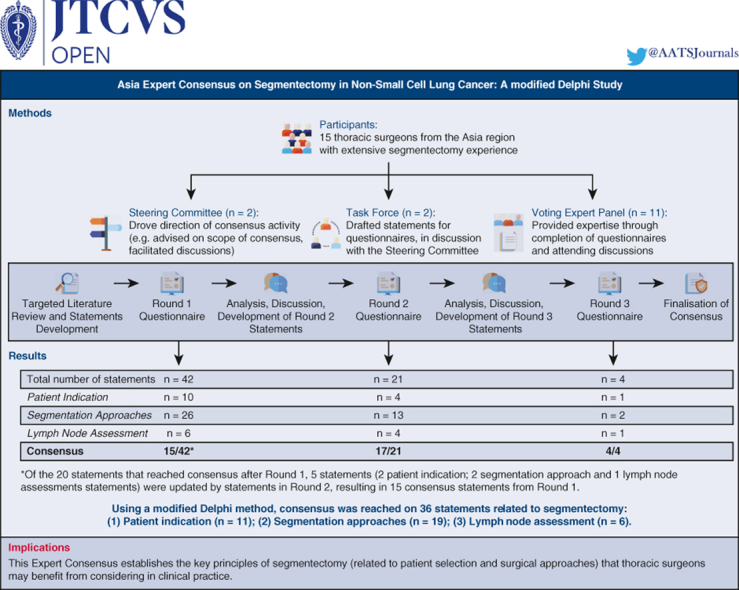

**Figure:**
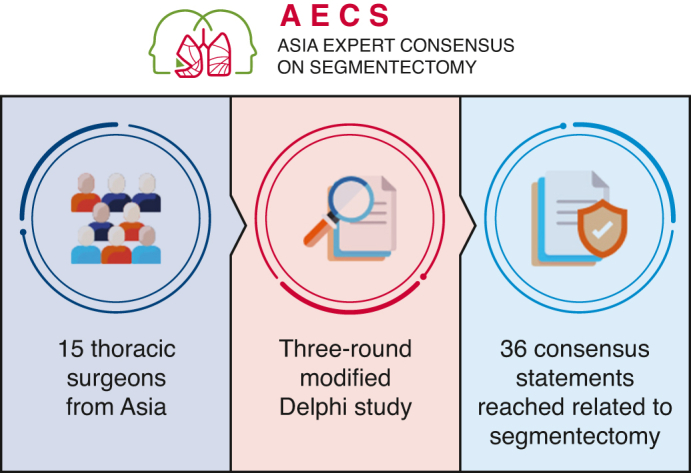
A modified Delphi approach was used to establish consensus on aspects of segmentectomy.

**Central Message**

This consensus is for surgeons considering segmentectomy in patients with early non–small cell lung cancer, outlining key principles of patient indication, surgical approaches, lymph node assessment.

**Perspective**

Segmentectomy is a parenchymal-sparing approach recommended over lobectomy in selected patients with early non–small cell lung cancer. A phase 3 trial reported improved 5-year overall survival for segmentectomy versus lobectomy, proposing thoracic surgeons to consider segmentectomy in suitable patients. This consensus addresses gaps in clinical guidance on patient selection and surgical approaches.

Lung cancer is the leading cause of cancer-related deaths in recent years and accounted for 18.0% of global cancer deaths in 2020.^1^ Approximately 1.3 million new lung cancer cases were diagnosed in 2020 in Asia-Pacific,^1^ and non–small cell lung cancer (NSCLC) accounts for approximately 81.3% of all cases of lung cancer.^2^

Although lobectomy has been regarded as the gold standard surgical treatment for early-stage NSCLC, there has been increasing interest in the use of parenchymal-sparing surgery, including segmentectomy, in early-stage NSCLC.^3^ The 2020 National Comprehensive Cancer Network Guidelines states that anatomic pulmonary resection is preferred for most patients; segmentectomy should achieve parenchymal margins ≥2 cm than the size of the nodule; segmentectomy should also sample appropriate N1 and N2 lymph node stations unless not technically feasible without substantially increasing the surgical risk; segmentectomy is appropriate in patients who have poor pulmonary reserve or other major comorbidity that contraindicates lobectomy or a peripheral nodule ≤2 cm with either pure adenocarcinoma in situ histology, ≥50% ground-glass opacity (GGO) appearance on computed tomography, or radiologic surveillance confirms a long doubling time of ≥400 days.^3^

More recently, results from a phase 3 randomized controlled trial (RCT), the Japan Clinical Oncology Group 0802 (JCOG0802) comparing segmentectomy against lobectomy in patients with invasive peripheral NSCLC tumor of diameter ≤2 cm and consolidation/tumor ratio >0.5 provides evidence of segmentectomy's longer-term safety and efficacy, suggesting the value of segmentectomy over lobectomy in these patients.^4^ The JCOG0802 RCT reported significant improvement in 5-year overall survival rates for segmentectomy compared with lobectomy (94.3% vs 91.1%; hazards ratio, 0.66; 95% confidence interval, 0.474-0.927; one-sided *P* < .0001 for noninferiority and *P* = .0082 for superiority).^4^ The 5-year relapse-free survival rates were comparable between groups (segmentectomy: 88.0% vs lobectomy: 87.9%; hazard ratio, 0.998; 95% confidence interval, 0.753-1.323).^4^

Despite the growing body of evidence supporting the role of segmentectomy in the surgical management of patients with early-stage NSCLC, there is limited clinical guidance on selecting patients who are best suited for segmentectomy. In particular, there are limited guidelines on the use of segmentectomy for different tumor types (eg, pure-solid vs solid-dominant or GGO dominant). In addition, the experience from Asia may be different from the National Comprehensive Cancer Network guidelines. Lastly, approaches to segmentectomy have been reported to vary, largely due to the procedure's complex nature.^5^

The objective of this study was to develop consensus on 3 aspects of segmentectomy (patient indication, segmentation approaches, and lymph node assessment) from an Asian perspective, which may guide thoracic surgeons on the use of segmentectomy in patients with early-stage NSCLC.

## Methods

A modified Delphi method was used to develop the Asia Expert Consensus on Segmentectomy (AECS). Ethical approvals were not applicable to this study, as this consensus is based on published literature and does not contain any new studies conducted on human research subjects or animals performed by any of the authors.

### Expert Selection

Fifteen thoracic surgeons representing Asia with extensive segmentectomy experience were invited to form the AECS Working Group. There were 3 roles among the AECS Working Group, which were the Steering Committee, Task Force, and Expert Panel. Two experts were part of the Steering Committee, 2 experts were part of the Task Force, and 11 experts were part of the voting Expert Panel (Table 1). All participating experts are authors of this article.

**Table 1:** Asia Expert Consensus on Segmentectomy working group members and roles

Title and name	Role
Dr Lunxu Liu	Steering Committee
Dr Shun-ichi Watanabe	Steering Committee
Dr Chengwu Liu	Task Force Member
Dr Masaya Yotsukura	Task Force Member
Dr Chang Chen	Voting (Expert Panel)
Dr Chun Chen	Voting (Expert Panel)
Dr Lijie Tan	Voting (Expert Panel)
Dr Liang Chen	Voting (Expert Panel)
Dr Kenji Suzuki	Voting (Expert Panel)
Dr Keiju Aokage	Voting (Expert Panel)
Dr Wataru Nishio	Voting (Expert Panel)
Dr Chang Young Lee	Voting (Expert Panel)
Dr Yong Hee Kim	Voting (Expert Panel)
Dr Chia-Chuan Liu	Voting (Expert Panel)
Dr Yau-Lin Tseng	Voting (Expert Panel)

As part of the Steering Committee, surgeons representing leading academic affiliations led the direction of the consensus activity, including advising on scope of consensus, facilitating discussions at the expert meeting, and finalizing all statements. The Task Force was nominated by the Steering Committee to support the review of evidence and to draft statements in discussion with the Steering Committee. The Steering Committee and Task Force did not participate in voting. Only the Expert Panel participated in voting. All surgeons from the AECS Working Group participated in hybrid (in-person and virtual) meetings that provided a platform for the experts to exchange insights and provide suggestions on statements.

Surgeons were eligible to join the AECS Working Group if they met the following criteria: had extensive experience with segmentectomy (≥100 cases); were a member of a professional organization or authority (ie, affiliations); were fluent in English, were willing to share their knowledge and experience; and had time to commit to attendance at meetings and to complete premeeting and postmeeting readings. Additional selection criteria were applicable for experts in the Steering Committee: highly recognized in the field in Asia; a strong record of contributing to discussions at forums, demonstrating thought leadership; confident and proactive in providing constructive and critical feedback in English; and able to commit the required time.

### Statement Development and Literature Review

Topics for the consensus were prioritized by the Steering Committee to be the most relevant to the objective based on clinical experience. Statements were grouped into 3 main topics: patient indication, segmentation approaches, and lymph node assessment. Statements regarding the topic of segmentation approaches were categorized into subtopics: preoperative planning; techniques of segmentectomy; recognition of intersegmental plane or intraoperative identification of the target segment; types of segmentectomy; surgical devices for making an intersegmental plane; ligation of pulmonary vasculature; prevention and management of air leaks; and surgeon considerations. Given the broad scope of the segmentation approaches topic, the Steering Committee chose to identify topics that were most essential to which a relatively inexperienced surgeon to be aware.

**Figure E1:**
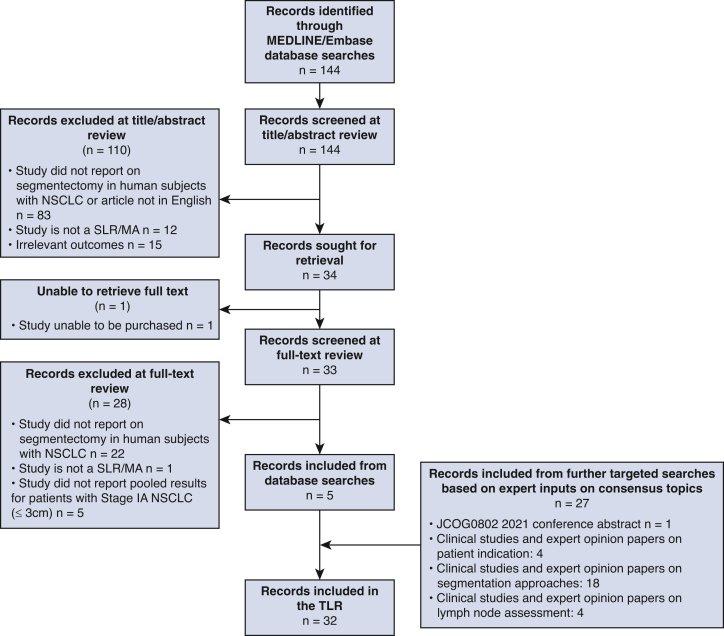
Flowchart of TLR records, A Preferred Reporting Items for Systematic Reviews and Meta-Analyses flowchart detailing the flow of study records through the different phases of the TLR. It maps out the number of records identified, included/excluded, and the reasons for exclusions. *NSCLC*, Non–small cell lung cancer; *SLR*, systematic literature review; *MA*, meta-analysis; *TLR*, targeted literature review.

A targeted literature review (TLR) was conducted to identify evidence on selected topics in segmentectomy, which was used to stimulate expert thinking (Figure E1). An initial search was performed, involving searching electronic scientific databases (MEDLINE and Embase databases via the Ovid Platform) to identify the most recent, highest-quality systematic literature reviews (SLRs) and meta-analyses summarizing the clinical and/or economic evidence for segmentectomy as compared with lobectomy in NSCLC. Table E1 lists the search terms used in the database searches. Additional targeted searches were conducted to identify relevant evidence as new statements were drafted, reviewed, and voted on. This pragmatic, iterative approach was deemed sufficient by the Steering Committee, considering that the aim of the modified Delphi study was to capture areas of consensus among experts, based on their knowledge of the topics and their individual clinical practice. All included references were reviewed by the Expert Panel over the 3 Delphi rounds. The output from the TLR was used in conjunction with individual clinical experiences to inform the statements drafted by the Steering Committee and Task Force.

### Delphi Rounds

For each Delphi round, the statements and references were distributed to the Expert Panel by e-mail and all voting and feedback were collected in Microsoft Office Forms. The Expert Panel were asked to vote anonymously on a 5-point Likert scale (1—Strongly Disagree to 5—Strongly Agree), and were given an opportunity to review, feedback, comment on those statements they disagreed or were neutral about and provide alternative references (if applicable) through open-text fields. Statements met consensus if ≥70% of respondents voted either “Agree”/“Strongly Agree” or “Disagree”/“Strongly Disagree.”

The Steering Committee also facilitated a group interaction among the Working Group to collectively review and comment on the outcomes after every round of the Delphi Panel through a face-to-face or virtual meeting (as permitted by the local conditions during the coronavirus disease 2019 pandemic). All experts (Steering Committee, Task Force, Expert Panel) reviewed the statements that met consensus, and discussed suggested changes and comments received (if any) for statements that did not reach consensus. Statements that did not reach consensus were then reworded, consolidated, or completely removed. All updated statements were then finalized by the Steering Committee and Task Force before being put up for the next round of voting. The Working Group collaborated with a third-party health care consultancy (Costello Medical) that coordinated and anonymized all responses so that all experts were blinded from each other's inputs.

## Results

All 11 experts in the Expert Panel completed all 3 Delphi rounds. There were no missing answers in this study. At the end of 3 Delphi rounds, 36 statements reached consensus, of which 11 were on patient indication, 19 were on segmentation approaches, and 6 were on lymph node assessment (Table 2).

**Table 2:** List of statements that reached consensus

S/N	Statement
Patient indication	
1.	In deciding the suitability of segmentectomy in patients with stage 1A NSCLC, it is appropriate to consider a combination of tumor diameter, consolidation/tumor ratio, and SUVmax values.
2.	Compromised segmentectomy may be one of the options in patients with advanced age, poor performance status, or poor cardiopulmonary reserve.
3.	Segmentectomy can achieve comparable postoperative 5-y overall survival vs lobectomy in selected patients.
4.	The greater risk of local recurrence associated with segmentectomy compared with lobectomy may be related to tumor characteristics, surgical margin, and extent of lymph node dissection; further evidence is needed to confirm this.
5.	Segmentectomy can better preserve postoperative respiratory function than lobectomy.
6.	Further evidence is needed to confirm the impact of segmentectomy on other patient outcomes, such as exercise capacity and quality of life.
7.	Segmentectomy can be considered in patients with peripheral stage IA NSCLC, where pure-solid tumors are ≤1 cm in diameter.
8.	Segmentectomy may be considered in patients with peripheral stage IA NSCLC, where pure-solid tumors are 1 to 2 cm in diameter.
9.	Segmentectomy can be considered in patients with peripheral stage IA NSCLC, where tumors with GGO (or nonsolid tumors) are ≤2 cm in total diameter.
10.	Segmentectomy may be considered in patients with peripheral stage IA NSCLC, where GGO-dominant tumors are 2 cm to 3 cm in total diameter.
11.	Shared decision-making is important before choosing sublobar resection or lobectomy for clinical stage I lung cancer. The surgeon should work with patient on current evidence-based information, the surgeon’s experience, and the patient’s preferences.
Segmentation approaches (preoperative planning)	
12.	Preoperative planning includes identification of the nodule and determining which segment should be dissected.
13.	Before the operation, the target segmental bronchus and pulmonary vasculatures should be identified by carefully reviewing the CT images or 3D model.
Segmentation approaches (techniques of segmentectomy)	
14.	Segmentectomy can be proceeded from hilum or interlobar fissures considering location of segments and degree of fissure development. For more complex anatomical basal segmentectomy, one can proceed through the inferior pulmonary ligament approach and use the method of stem-branch to track the anatomy.
Segmentation approaches (recognition of intersegmental plane or intraoperative identification of the target segment)	
15.	Techniques to identify the intersegmental planes and/or resection lines include: differential ventilation (inflation/deflation) after identification of the target bronchus, intravenous ICG injection with the pulmonary artery supplying the target segment divided or temporarily clamped, identification of the intersegmental pulmonary vein, differential dyeing using endobronchial dye injection, and lung surface intersegmental constant proportion landmarks.
Segmentation approaches (types of segmentectomy)	
16.	Surgeons with no previous experience with segmentectomy should gain experience with simple segmentectomy first before performing complex and/or combined segmentectomies.
17.	Segmentectomy can be classified into 2 categories: simple segmentectomy and complex segmentectomy.
18.	Simple segmentectomy forms a single intersegmental plane, such as resections of the superior segment, left upper division, lingular segmentectomies, and entire basal segment of the lower lobe.
19.	Complex segmentectomy forms ≥2 intersegmental planes, including single segment resection of RS1, RS2, RS3, RS7, RS8, RS9, RS10, LS1+2, LS3, LS8, LS9, LS10.
20.	Combined segmentectomy is a combination of ≥2 segments and/or sub-segments resection (eg, S8+9, S9+10, RS2a+3b, RS1a+2, and so on).
21.	A purpose of combined segmentectomy is securing the segmental margin.
Segmentation approaches (surgical devices for making an intersegmental plane)	
22.	The choice of stapling devices or energy instruments for making an inter-segmental plane should consider: the risk of postoperative complications (eg, air leakage and pneumothorax, atelectasis), postoperative pulmonary function, local control for lung cancer, and prognosis.
23.	Compared with staplers, the use of energy devices alone (eg, electrocautery, ultrasonic scalpel) may result in a higher rate of post-operative air leaks.
24.	Complex segmentectomy may require more endoscopic stapler firings compared with lobectomy due to the need for longer transection lines over intersegmental planes.
Segmentation approaches (ligation of pulmonary vasculature)	
25.	Advanced energy-based devices (eg, ultrasonic dissectors) and vascular staplers may be a suitable alternative to conventional methods for ligation (eg, suture ligation, vascular clips) of pulmonary vasculature in segmentectomy.
Segmentation approaches (prevention and management of air leaks)	
26.	A preventive measure for reducing the risk of prolonged air leaks is following the right pathway of the intersegmental plane.
27.	Intraoperative inflation of the residual lung under water seal is crucial for detecting air leaks after segmentectomy.
28.	Prolonged air leak is one of the most common complications associated with segmentectomy and is associated with greater morbidity rates.
29.	Preventive measures for reducing the risk of prolonged air leaks include: following the right pathway of the intersegmental plane, the use of sealants and buttressing staple lines.
Segmentation approaches (surgeon considerations)	
30.	Minimally invasive (video-assisted) complex segmentectomy is more challenging to perform than minimally invasive (video-assisted) lobectomy.
Lymph node assessment	
31.	Intralobar lymph nodes should be assessed or sampled during segmentectomy.
32.	Interlobar lymph nodes should be assessed or sampled during segmentectomy.
33.	Hilar lymph nodes should be assessed or sampled during segmentectomy for solid-dominant tumors.
34.	Mediastinal lymph nodes should be assessed or sampled during segmentectomy for solid-dominant tumors.
35.	Evidence supports the use of advanced energy-based devices (eg, ultrasonic dissectors) for lymphadenectomy in lobectomy—based on clinical experience, the efficacy of advanced energy-based devices for lymphadenectomy in segmentectomy could be similar.
36.	The significance of lymph node dissection in patients with stage 1 NSCLC of solid tumor undergoing segmentectomy is undefined.

*S/N*, Statement number; *NSCLC*, non–small cell lung cancer; *SUVmax*, maximum standard unit value; *GGO*, ground-glass opacity; *CT*, computed tomography; *3D*, 3-dimensional; *ICG*, intravenous indocyanine green.

**Figure 1:**
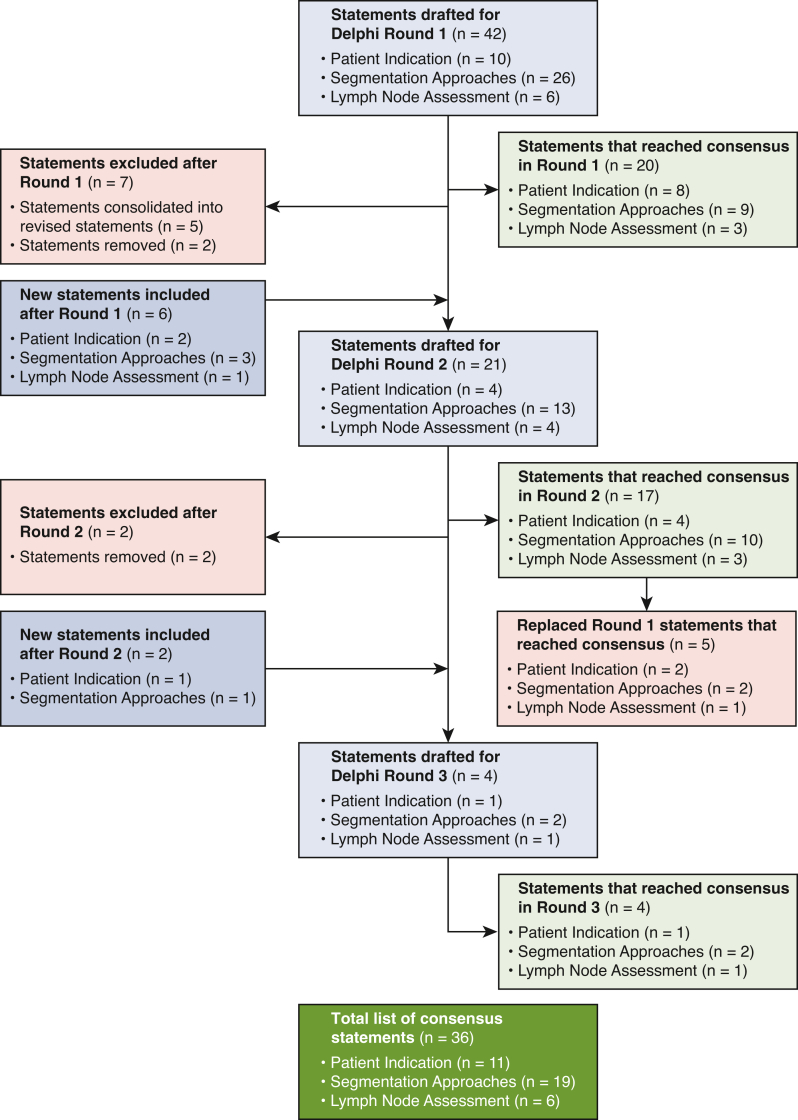
Summary of results. A flowchart detailing the total number of statements that were drafted; reached consensus; newly added; removed; or replaced over the 3 Delphi rounds.

**Figure 2:**
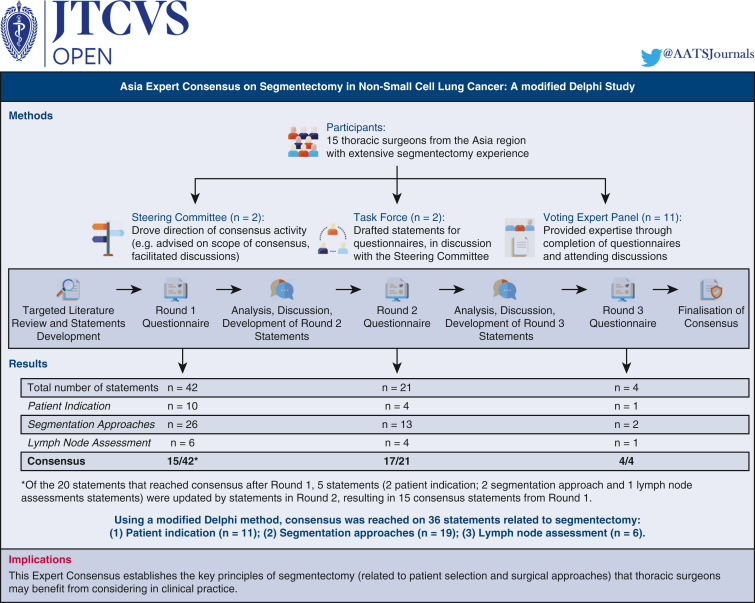
Graphical abstract of the study.

A summary of results can be found in Figure 1. Please refer to Figure 2 for the Graphical Abstract of the study's methods, results, and implications. An audio–visual summary of this study is included as Video 1.

### Round 1

A summary of the TLR can be found in Figure E1. The Steering Committee and Task Force drafted 42 statements for Round 1. There were 10 statements on Patient Indication, 26 statements on Segmentation Approaches, and 6 statements on Lymph Node Assessment. By the end of Round 1, 20 of the 42 (48%) drafted statements reached consensus; the remaining 22 statements did not reach consensus (Table E2).

### Round 2

After Round 1, 11 statements were revised, 4 statements were revoted, 5 statements were consolidated into the revised statements, and 2 statements were removed. Finally, 6 new statements were drafted. In round 2, there were 4 statements on patient indication, 13 statements on segmentation approaches, and 4 statements on lymph node assessment. By the end of round 2, 17 of the 21 (81%) drafted statements reached consensus, whereas 4 statements did not reach consensus (Table E3). In addition, 5 statements that reached consensus in round 1 were updated by statements in round 2 (Table E4).

### Round 3

After round 2, 2 statements were revised, 2 statements were removed, and 2 new statements were drafted. In round 3, there was 1 statement on patient indication, 2 statements on segmentation approaches, and 1 statement on lymph node assessment. By the end of round 3, 4 of the 4 (100%) drafted statements reached consensus (Table E5).

## Discussion

This study established the key principles related to segmentectomy in NSCLC by developing 36 expert consensus statements, based on a combination of the experience of thoracic surgery experts in Asia as well as published literature, to address existing gaps in clinical guidelines.

### Patient Indication

Historically, evidence supporting the role of segmentectomy in NSCLC has come from observational studies reporting noninferior survival outcomes for segmentectomy compared with lobectomy.^6^^,^^7^ Recently, positive long-term clinical outcomes for segmentectomy compared with lobectomy reported in the JCOG0802 RCT contribute to the growing evidence base on segmentectomy as a parenchymal-sparing surgery in early NSCLC and highlight a need to consolidate current understanding on the patient or oncologic factors that could enable successful outcomes with segmentectomy.^4^ To date, a range of tumor and patient factors have been reported as being important when determining a patient's suitability for segmentectomy; these include the size, location, and characteristics of tumor, as well as patient's clinical status.^8^ Experts reached consensus on several statements (#3-5, #7-9) based on these data and their clinical experience, and is aligned to a previous expert opinion paper discussing patient selection for segmentectomy, which had suggested segmentectomy in tumors ≤2 cm.^9^ Experts agreed that patients with peripheral GGO-dominant tumors of ≤3 cm may be suitable for segmentectomy, which reflects the difference in malignancy risk as compared with solid-tumors.

### Segmentation Approaches

Variations in segmentation techniques were reflected in published expert reviews reporting on individual or institutional segmentation approaches.^5^^,^^10^

#### Preoperative planning

Considering that surgical decision-making is highly dependent on tumor characteristics,^4^^,^^6^^,^^7^ preoperative planning is an essential step to better understand the patient's condition and to assess whether segmentectomy is suitable. Experts agreed that preoperative planning includes identification of the nodule and determining which segment(s) should be dissected using computed tomography images and 3-dimensional models. Specific attention may be paid to the anatomy and relative positioning of the basal segmental vessels and bronchi, target nodules and surrounding anatomic structures, as well as the lung surface state, when informing the surgical excision approach.^11^ Experts noted that a key goal when planning the extent of segmentectomy is ensuring sufficient surgical margin can be achieved as insufficient surgical margins may be associated with the risk of locoregional recurrence.^12^

#### Techniques of segmentectomy

Experts agreed that segmentectomy can be proceeded from hilum or interlobar fissures considering location of segments and degree of fissure development. For more complex anatomical basal segmentectomy, one can proceed through the inferior pulmonary ligament approach and use the method of stem-branch to track the anatomy. While a specific segmentectomy technique reached consensus, it is not exhaustive and alternative techniques may be developed in the future as more surgeons gain experience with segmentectomy.

#### Recognition of intersegmental plane or intraoperative identification of the target segment

There are various methods available to identify the intersegmental plane or the target segment. Consistent with published literature,^5^^,^^13^ experts agreed that techniques to identify the intersegmental planes and/or resection lines include differential ventilation (inflation/deflation) after identification of the target bronchus, intravenous indocyanine green (ICG) injection with the pulmonary artery supplying the target segment divided or temporarily clamped, identification of the intersegmental pulmonary vein, differential dyeing using endobronchial dye injection, and lung surface intersegmental constant proportion landmarks. No technique was agreed to be particularly advantageous over another.

Differential ventilation has been reported to be relatively easy to perform and is commonly used. However, the inflation/deflation border may be unclear due to collateral ventilation,^5^ depending on the degree of emphysema, which can potentially also affect operation time. Intravenous ICG injection is similarly an efficient technique and may provide more accurate demarcation of the intersegmental plane, as it shows a more restricted intersegmental plane compared with differential ventilation.^13^ Intravenous ICG may be injected into the resected bronchus during the operation.^14^ Accessory bronchial branches may, however, lead to incorrect intravenous ICG stains in endobronchial dye injection.^5^

An additional consideration in the identification of intersegmental planes is the pulmonary bronchial vasculature, which forms the boundaries of pulmonary segments. A good understanding of the variations in the pulmonary bronchial vasculature, especially the branching patterns of pulmonary veins, supplements any imaging performed during preoperative planning, and also the aforementioned techniques for identifying the intersegmental planes.^15^ Different branching patterns exist, which can complicate application of this method in practice.^16^

Experts agreed that “lung surface intersegmental constant proportion landmarks” may also be a way to recognize the intersegmental plane. The lack of specific surface projections on the lung, apart from pseudo-fissures, presents a challenge to using morphological landmarks to identify the intersegmental plane. In one study, the lengths of specific segments along the lobe's anatomic landmark lines were measured and found to be a constant proportion, suggesting that lung surface intersegmental constant proportion landmarks could facilitate identification of the intersegmental planes during anatomic segmentectomy.^17^ Further investigation is needed to confirm the validity of this technique.

#### Types of segmentectomy

No formal framework for classifying segmentectomy by anatomy or complexity has been established to date, although some definitions have been suggested in literature.^18^ Experts agreed that segmentectomy can be classified as “simple,” “complex,” or “combined,” where the purpose of combined segmentectomy is to secure the segmental margin beyond the conventional anatomical segments.

Studies that compared the technical (operative time) and clinical (safety, efficacy) aspects of simple versus complex segmentectomies present a mixed picture. Although one study reported that complex segmentectomy requires significantly longer operation time compared with simple segmentectomy (180 vs 143.5 minutes, *P* < .0001),^18^ another study reported equivalent median operative time.^19^ In one study, 30-day mortality rates, complication rates, median surgical margin distance, and number of dissected lymph nodes were comparable between groups.^18^ However, another study reported fewer postoperative complications with complex segmentectomy.^19^ Further evidence is needed to confirm the relative safety and efficacy of simple versus complex segmentectomy.

#### Surgical devices for making an intersegmental plane

An SLR comparing electrocautery versus stapler in segmentectomy reported that electrocautery resulted in a greater incidence rate of air leaks.^20^ Experts agreed that compared with staplers, the use of energy devices alone (eg, electrocautery, ultrasonic scalpel) may result in a greater rate of postoperative air leaks. Energy devices may result in some heat damage to lung tissues, which may give rise to postoperative complications such as air leaks.^20^

#### Prevention and management of air leaks

It is well-established that prolonged air leak are one of the most common potential complications associated with segmentectomy and are associated with greater morbidity rates.^21^ It is worth noting that air leaks are not clinically more significant in segmentectomy than in lobectomy: a retrospective database study reported that patients who underwent segmentectomy had lower odds of developing air leaks compared with patients who underwent lobectomy (odds ratio, 0.77; 95% confidence interval, 0.67-0.89).^22^ Experts agreed that following the right pathway of the intersegmental plane is important periprocedure to prevent air leaks.

#### Surgeon considerations

Experts agreed that minimally invasive video-assisted complex segmentectomy is more challenging to perform than minimally invasive video-assisted lobectomy. Published literature reported similar conclusions, citing the need for 3-dimensional knowledge of the segmental bronchovascular relationships and possible anatomical variations for complex segmentectomy.^23^ As an extension of this, experts commented that potential challenges are identifying the segmental pulmonary vessels and bronchus; positioning the mechanical stapler appropriately, given that there are multiple intersegmental borders in complex segmentectomy; likewise, securing sufficient surgical margins requires great surgical skill, particularly when using a minimally invasive approach to perform complex segmentectomy.

### Lymph Node Assessment

There is an ongoing debate on the adequacy of lymph node sampling, as well as the role of lymph node assessment in segmentectomy, due to limited evidence on recurrence risk, nodal upstaging, and the reasonable extent of dissection.^24^^,^^25^ Kodia and colleagues^26^ suggest that improvements in overall survival associated with lobectomy may potentially be confounded by the adequacy of lymph node harvest in lobectomy compared with segmentectomy, leading to the question of whether segmentectomy may have equivalent oncologic outcomes to lobectomy if adequate lymph node dissection can be achieved.

Ultimately, preoperative imaging is likely limited in detecting lymph node metastasis, so lymph node dissection may be needed for accurate staging. Our consensus reported that experts agreed intralobar and interlobar lymph nodes should be at least assessed or sampled during segmentectomy. In addition, experts also agreed that hilar or mediastinal lymph nodes should be at least assessed or sampled in solid-dominant tumors. This is due to the increased malignancy risk of solid-dominant tumors, which tend to have more nodal involvement compared with subsolid tumors.^27^^,^^28^ The location of segmentectomy may also affect the risk of nodal recurrence: small peripheral lower-lobe tumors tend to metastasize to the lower mediastinal lymph nodes, which is associated with increased recurrence rates and poor survival.^25^ Results of the JCOG0802 RCT further suggest that more research is needed to investigate lymph node dissection and risk of relapse—despite hilar and mediastinal lymph node dissection being performed in all patients who underwent segmentectomy, ipsilateral or contralateral mediastinal lymph node relapse occurred more frequently in the segmentectomy group than the lobectomy group.^4^

### Gaps and Areas of Nonconsensus

Experts were not able to reach consensus on specific procedural strategies or techniques for segmentectomy (Table E3), which suggests the variability in surgical techniques used among experts. Experts also discussed that the hilar and mediastinal lymph nodes should be assessed or sampled only for solid-dominant tumors (Table 2) because evidence regarding other tumor subtypes is currently unclear. Further studies could be conducted to address additional aspects of segmentectomy (eg, specific techniques, lymph node assessment in varying tumor types, segmentectomy vs wedge resection) when more evidence is available.

### Strengths and Limitations

The strength of the Delphi approach lies in the use of a structured approach to elicit expert views on a topic of debate, allowing the assessment of the extent of agreement and to resolve disagreements on a topic.^29^ It has been used extensively to establish consensus across a range of scientific areas, even in the field of thoracic surgery.^30^

This study has limitations as follows: A pragmatic approach was taken in the literature review, wherein published SLRs were leveraged to gain an understanding of the available evidence base, in an efficient manner. By design, Delphi studies are driven by expert opinion that is subjective and may differ from that of other surgeons. We also acknowledge that the voting group of 11 experts is relatively small and that a larger panel of experts would increase the robustness of the results; however, this study aimed to achieve reliable outcomes, with minimal bias, by including more than 10 voting experts with extensive experience in segmentectomy from 4 Asia regions, representing diverse health care systems and clinical practices. The statements presented in this document may be a reflection of the cumulative experiences of a group of experts in Asia and may not necessarily be generalizable to other geographical regions where the biology of the tumor could differ. In addition, we would like to highlight that this study is based on current knowledge and experience, which may evolve over time as segmentectomy is adopted more widely.

## Conclusions

The recent JCOG0802 RCT reported significantly improved 5-year overall survival rates for segmentectomy versus lobectomy, proposing thoracic surgeons to consider segmentectomy as a surgical option in suitable patients. This consensus serves as a guidance to thoracic surgeons considering segmentectomy in patients with early NSCLC, outlining key principles related to patient indication, segmentectomy approaches and lymph node assessment that surgeons should consider in surgical decision-making.

### Conflict of Interest Statement

Medical writing support was provided by Costello Medical Singapore. LL: J&J provided an honorarium for the participation and contribution to the Delphi phase of the study. AK: J&J provided an honorarium for the participation and contribution to the Delphi phase of the study. CL: J&J provided an honorarium for the participation and contribution to the Delphi phase of the study. KYH: J&J provided an honorarium for the participation and contribution to the Delphi phase of the study. LCY: J&J provided an honorarium for the participation and contribution to the Delphi phase of the study. LC: J&J provided an honorarium for the participation and contribution to the Delphi phase of the study. NW: J&J provided an honorarium for the participation and contribution to the Delphi phase of the study. SK: J&J provided an honorarium for the participation and contribution to the Delphi phase of the study. TL: J&J provided an honorarium for the participation and contribution to the Delphi phase of the study. TYL: J&J provided an honorarium for the participation and contribution to the Delphi phase of the study. YM: J&J provided an honorarium for the participation and contribution to the Delphi phase of the study. WS: J&J provided an honorarium for the participation and contribution to the Delphi phase of the study. All other authors reported no conflicts of interest.

The *Journal* policy requires editors and reviewers to disclose conflicts of interest and to decline handling or reviewing manuscripts for which they may have a conflict of interest. The editors and reviewers of this article have no conflicts of interest.

## Figures and Tables

**Table E1:** TLR search terms

Term group	#	Search terms	Results
NSCLC and surgery	1.	NSCLC.ti,ab,kw,kf.	139,809
	2.	exp Carcinoma, Non-Small-Cell Lung/or non small cell lung cancer/	171,490
	3.	(lung$ and (non small cell or nonsmall cell) and (carcinoma$ or adenocarcinoma$ or cancer$ or tumo?r$ or neoplasm$)).ti,ab,kw,kf.	190,265
	4.	or/1-3	259,924
	5.	exp Thoracic Surgery/or exp Thoracic Surgical Procedures/or exp Thorax Surgery/	984,361
	6.	((lobectom$ or segment$) adj4 (pulmonar$ or lung$ or thoracic$)).ti,ab,kw,kf.	26,439
	7.	((((resect$ or surger$ or excis$ or surgic$ or remov$) adj2 (pulmonar$ or lung$ or thoracic$ or sublobar$)) or ((resect$ or surger$ or excis$ or surgic$ or remov$) adj3 lobe$)) and (pulmonar$ or lung$)).ti,ab,kw,kf.	83,990
	8.	exp Thoracic Surgery, Video-Assisted/or exp video assisted thoracoscopic surgery/	21,070
	9.	("video assisted thoracic surg$" or VATS or "robot assisted thoracic surg$").ti,ab,kw,kf.	18,192
	10.	or/5-9	1,040,271
	11.	4 and 10	27,489
SLRs and MAs	12.	Meta-Analysis as Topic/	53,597
	13.	meta analy$.tw.	469,041
	14.	metaanaly$.tw.	13,002
	15.	exp Meta-Analysis/	357,868
	16.	(systematic adj (review$1 or overview$1)).tw.	468,401
	17.	exp Review Literature as Topic/	252,291
	18.	cancerlit.ab.	1371
	19.	cochrane.ab.	228,966
	20.	embase.ab.	252,910
	21.	(psychlit or psyclit).ab.	1917
	22.	(psychinfo or psycinfo).ab.	84,499
	23.	(cinahl or cinhal).ab.	73,724
	24.	science citation index.ab.	7141
	25.	bids.ab.	1294
	26.	cancerlit.ab.	1371
	27.	reference list$.ab.	42,415
	28.	bibliograph$.ab.	44,647
	29.	hand-search$.ab.	16,677
	30.	relevant journals.ab.	2724
	31.	manual search$.ab.	10,909
	32.	or/12-31	1,178,180
	33.	selection criteria.ab.	71,785
	34.	data extraction.ab.	55,174
	35.	33 or 34	122,144
	36.	Review/or review.pt.	5,678,018
	37.	35 and 36	62,308
	38.	32 or 37	1,187,887
Exclusion terms	39.	("conference abstract" or "conference review").pt.	4,140,588
	40.	exp animals/not exp humans/	9,664,462
	41.	(comment or editorial or case reports or historical article).pt.	4,530,118
	42.	comment/or editorial/or case reports/	4,163,280
	43.	(case stud$ or case report$).ti.	717,224
	44.	historical article/	364,384
	45.	case study/	2,273,414
	46.	or/39-45	18,349,853
Combination and total	47.	11 and 38	861
	48.	47 not 46	685
	49.	remove duplicates from 48	517
	50.	limit 49 to yr = "2018-current"	144

Database(s): Ovid MEDLINE(R) and Epub Ahead of Print, In-Process, In-Data-Review & Other Non-Indexed Citations and Daily (via OVID SP; 1974-12 July 2021); Embase (via OVID SP; 1974-12 July 2021). *NSCLC*, Non–small cell lung cancer; *VATS*, video-assisted thoracoscopic surgery; *SLR*, systematic literature review; *MA*, meta-analysis.

**Table E2:** Results from round 1 Delphi (42 statements)

Statement	Percentage of experts who strongly agree/agree	Percentage of experts who strongly disagree/disagree	Consensus achieved
Patient indication			
Segmentectomy can be considered in patients with stage IA NSCLC, where pure-solid tumors are ≤1 cm in diameter.	81.82%	9.09%	Yes
Segmentectomy may be considered in patients with stage IA NSCLC, where pure-solid tumors are ≤2 cm in diameter.	45.45%	9.09%	No
Segmentectomy should be considered in patients with stage IA NSCLC, where tumors with GGO (or nonsolid tumors) are ≤2 cm in diameter.	63.64%	27.27%	No
Segmentectomy may be considered in patients with stage IA NSCLC, where GGO-dominant tumors are ≤3 cm in diameter.	81.82%	0.00%	Yes
In deciding the suitability of segmentectomy in patients with stage 1A NSCLC, it is appropriate to consider a combination of tumor diameter, consolidation/tumor ratio and SUVmax values.	81.82%	9.09%	Yes
Compromised segmentectomy may be one of the options in patients with advanced age, poor performance status, or poor cardiopulmonary reserve.	90.91%	9.09%	Yes
Segmentectomy can achieve comparable postoperative 5-y overall survival vs lobectomy in selected patients.	90.91%	9.09%	Yes
The greater risk of local recurrence associated with segmentectomy compared with lobectomy may be related to tumor characteristics, surgical margin, and extent of lymph node dissection; further evidence is needed to confirm this.	90.91%	9.09%	Yes
Segmentectomy can better preserve postoperative respiratory function than lobectomy.	72.73%	9.09%	Yes
Further evidence is needed to confirm the impact of segmentectomy on other patient outcomes, such as exercise capacity and quality of life.	90.91%	9.09%	Yes
Segmentation approaches			
Preoperative planning includes identification of the nodule and determining which segment should be dissected.	100.00%	0.00%	Yes
Before the operation, the target segmental bronchus and pulmonary vasculatures should be identified by carefully reviewing the CT images or 3D model.	100.00%	0.00%	Yes
A technique to identify the intersegmental planes and/or resection lines is differential ventilation (inflation/deflation) after identification of the target bronchus.	63.64%	0.00%	No
A technique to identify the intersegmental planes and/or resection lines is intravenous ICG injection with the pulmonary artery supplying the target segment divided or temporarily clamped.	63.64%	0.00%	No
A technique to identify the intersegmental planes and/or resection lines is identification of the intersegmental pulmonary vein.	81.82%	0.00%	Yes
A technique to identify the inter-segmental planes and/or resection lines is differential dyeing using endobronchial dye injection.	18.18%	27.27%	No
A technique to identify the intersegmental planes and/or resection is lung surface intersegmental constant proportion landmarks.	18.18%	18.18%	No
Segmentectomy can be classified into 3 categories: simple segmentectomy; complex segmentectomy; combined segmentectomy/subsegmentectomy.	54.55%	9.09%	No
Simple segmentectomy forms a single intersegmental plane, such as resections of the superior segment, left upper division, lingular segmentectomies and entire basal segment of the lower lobe.	63.64%	0.00%	No
Complex segmentectomy forms ≥2 intersegmental planes, including single segment resection of RS1, RS2, RS3, RS7, RS8, RS9, RS10, LS1+2, LS3, LS8, LS9, LS10.	54.55%	9.09%	No
Combined segmentectomy is a combination of ≥2 segments and/or subsegments resection (eg, S8+9, S9+10, RS2a+3b, RS1a+2, and so on).	63.64%	18.18%	No
A challenge in combined segmentectomy is securing the segmental margin.	63.64%	27.27%	No
Surgeons with no previous experience with segmentectomy should gain experience with simple segmentectomy first, before performing complex and/or combined segmentectomies.	72.73%	9.09%	Yes
The choice of stapling devices or energy instruments for making an intersegmental plane should consider: the risk of postoperative complications (eg, air leakage and pneumothorax, atelectasis), postoperative pulmonary function, local control for lung cancer, and prognosis.	81.82%	0.00%	Yes
Compared with staplers, energy devices (eg, electrocautery, ultrasonic scalpel) may result in a greater rate of postoperative air leaks and other complications (eg, hemorrhage, pneumonia, and pulmonary embolism).	63.64%	9.09%	No
Segmentectomy requires more endoscopic stapler firings compared with lobectomy due to the need for longer transection lines over multiple intersegmental planes.	63.64%	9.09%	No
Advanced energy-based devices (eg, ultrasonic dissectors) and vascular staplers may be a suitable alternative to conventional methods for ligation (eg, suture ligation, vascular clips) of pulmonary vasculature in segmentectomy.	81.82%	9.09%	Yes
Prolonged air leak is one of the most common complications associated with segmentectomy and is associated with increased length of postoperative hospital stay and greater morbidity rates.	63.64%	9.09%	No
A preventive measure for reducing the risk of prolonged air leaks is following the right pathway of the intersegmental plane.	72.73%	9.09%	Yes
A preventive measure for reducing the risk of prolonged air leaks is the use of sealants.	54.55%	0.00%	No
A preventive measure for reducing the risk of prolonged air leaks is buttressing staple lines.	45.45%	9.09%	No
A preventive measure for reducing the risk of prolonged air leaks is pleural tenting.	9.09%	54.55%	No
Intraoperative inflation of the residual lung under water seal is crucial for detecting air leaks after segmentectomy.	90.91%	0.00%	Yes
Another common complication associated with segmentectomy is atelectasis.	54.55%	18.18%	No
Minimally invasive (video-assisted) segmentectomy is more challenging to perform than minimally invasive (video-assisted) lobectomy.	81.82%	0.00%	Yes
There is minimal difference in the operation time for segmentectomy compared to lobectomy.	54.55%	27.27%	No
Lymph node assessment			
Intralobar lymph nodes should be assessed or sampled during segmentectomy.	81.82%	18.18%	Yes
Interlobar lymph nodes should be assessed or sampled during segmentectomy.	72.73%	18.18%	Yes
Hilar lymph nodes should be assessed or sampled during segmentectomy.	81.82%	9.09%	Yes
Mediastinal lymph nodes should be assessed or sampled during segmentectomy.	45.45%	18.18%	No
The significance of lymph node dissection in patients with early-stage NSCLC who are suitable for segmentectomy is unclear.	45.45%	18.18%	No
Evidence supports the use of advanced energy-based devices (eg, ultrasonic dissectors) for lymphadenectomy in lobectomy; the role of advanced energy-based devices for segmentectomy remains to be explored.	9.09%	54.55%	No

Neutral votes make up the remaining percentages for each statement. *NSCLC*, Non–small cell lung cancer; *GGO*, ground-glass opacity; *SUVmax*, maximum standard unit value; *CT*, computed tomography; *3D*, 3-dimensional; *ICG*, indocyanine green.

**Table E3:** Results from round 2 Delphi (21 statements)

Statement	Percentage of experts who strongly agree/agree	Percentage of experts who strongly disagree/disagree	Consensus achieved
Patient indication			
Segmentectomy can be considered in patients with peripheral stage IA NSCLC, where pure-solid tumors are ≤1 cm in diameter.	100.00%	0.00%	Yes
Segmentectomy may be considered in patients with peripheral stage IA NSCLC, where pure-solid tumors are 1 to 2 cm in diameter.	72.73%	9.09%	Yes
Segmentectomy can be considered in patients with peripheral stage IA NSCLC, where tumors with GGO (or nonsolid tumors) are ≤2 cm in total diameter.	100.00%	0.00%	Yes
Segmentectomy may be considered in patients with peripheral stage IA NSCLC, where GGO-dominant tumors are 2 to 3 cm in total diameter.	81.82%	0.00%	Yes
Segmentation approaches			
Techniques to identify the intersegmental planes and/or resection lines include: differential ventilation (inflation/deflation) after identification of the target bronchus, intravenous ICG injection with the pulmonary artery supplying the target segment divided or temporarily clamped, identification of the intersegmental pulmonary vein, differential dyeing using endobronchial dye injection, and lung surface intersegmental constant proportion landmarks.	90.91%	9.09%	Yes
An efficient procedure for segmentectomy is the method of “single-direction” technique, which enables exposure and dissection of the target segmental structures from superficial to deep in a simple manner.	27.27%	18.18%	No
A strategy for anatomical basal segmentectomy is to proceed through the inferior pulmonary ligament approach in a single-direction way and use the method of stem-branch to track the anatomy.	36.36%	18.18%	No
Segmentectomy can be classified into 2 categories: simple segmentectomy and complex segmentectomy.	90.91%	0.00%	Yes
Simple segmentectomy forms a single intersegmental plane, such as resections of the superior segment, left upper division, lingular segmentectomies and entire basal segment of the lower lobe.	90.91%	0.00%	Yes
Complex segmentectomy forms ≥2 intersegmental planes, including single segment resection of RS1, RS2, RS3, RS7, RS8, RS9, RS10, LS1+2, LS3, LS8, LS9, LS10.	81.82%	9.09%	Yes
Combined segmentectomy is a combination of ≥2 segments and/or subsegments resection (eg, S8+9, S9+10, RS2a+3b, RS1a+2, and so on).	72.73%	9.09%	Yes
A purpose of combined segmentectomy is securing the segmental margin.	90.91%	0.00%	Yes
Compared with staplers, the use of energy devices alone (eg, electrocautery, ultrasonic scalpel) may result in a greater rate of postoperative air leaks.	90.91%	0.00%	Yes
Segmentectomy requires more endoscopic stapler firings compared with lobectomy due to the need for longer transection lines over multiple intersegmental planes.	63.64%	9.09%	No
Prolonged air leak is one of the most common complications associated with segmentectomy and is associated with greater morbidity rates.	81.82%	0.00%	Yes
Preventive measures for reducing the risk of prolonged air leaks include: following the right pathway of the intersegmental plane, the use of sealants and buttressing staple lines.	90.91%	0.00%	Yes
Minimally invasive (video-assisted) complex segmentectomy is more challenging to perform than minimally invasive (video-assisted) lobectomy.	90.91%	0.00%	Yes
Lymph node assessment			
Hilar lymph nodes should be assessed or sampled during segmentectomy for solid-dominant tumors.	100.00%	0.00%	Yes
Mediastinal lymph nodes should be assessed or sampled during segmentectomy for solid-dominant tumors.	100.00%	0.00%	Yes
The significance of lymph node dissection in patients with stage 1 NSCLC of solid-tumor undergoing segmentectomy is unclear.	54.55%	27.27%	No
Evidence supports the use of advanced energy-based devices (eg, ultrasonic dissectors) for lymphadenectomy in lobectomy—based on clinical experience, the efficacy of advanced energy-based devices for lymphadenectomy in segmentectomy could be similar.	72.73%	9.09%	Yes

Neutral votes make up the remaining percentages for each statement. *NSCLC*, Non–small cell lung cancer; *GGO*, ground-glass opacity; *ICG*, indocyanine green.

**Table E4:** Updated statements

Subtopic	Statements after round 1 consensus∗	New statements after round 2 consensus
Patient indication	Segmentectomy can be considered in patients with stage IA NSCLC, where pure-solid tumors are ≤1 cm in diameter.	Segmentectomy can be considered in patients with peripheral stage IA NSCLC, where pure-solid tumors are ≤1 cm in diameter.
Patient indication	Segmentectomy may be considered in patients with stage IA NSCLC, where GGO-dominant tumors are ≤3 cm in diameter.	Segmentectomy may be considered in patients with peripheral stage IA NSCLC, where GGO-dominant tumors are 2 cm to 3 cm in total diameter.
Segmentation approaches (recognition of intersegmental plane or intra-operative identification of the target segment)	A technique to identify the inter-segmental planes and/or resection lines is identification of the intersegmental pulmonary vein.	Techniques to identify the inter-segmental planes and/or resection lines include: differential ventilation (inflation/deflation) after identification of the target bronchus, intravenous ICG injection with the pulmonary artery supplying the target segment divided or temporarily clamped, identification of the intersegmental pulmonary vein, differential dyeing using endobronchial dye injection, and lung surface intersegmental constant proportion landmarks.
Segmentation approaches (surgeon considerations)	Minimally invasive (video-assisted) segmentectomy is more challenging to perform than minimally invasive (video-assisted) lobectomy.	Minimally invasive (video-assisted) complex segmentectomy is more challenging to perform than minimally invasive (video-assisted) lobectomy.
Lymph node assessment	Hilar lymph nodes should be assessed or sampled during segmentectomy.	Hilar lymph nodes should be assessed or sampled during segmentectomy for solid-dominant tumors.

*NSCLC*, Non–small cell lung cancer; *GGO*, ground-glass opacity; *ICG*, intravenous indocyanine green.

∗ Of the 20 statements that reached consensus after round 1, 5 statements (2 patient indication; 2 segmentation approach and 1 lymph node assessment statements) were updated by statements in round 2, resulting in 15 consensus statements from round 1.

**Table E5:** Results from round 3 Delphi (4 statements)

Statement	Percentage of experts who strongly agree/agree	Percentage of experts who strongly disagree/disagree	Consensus achieved
Patient indication			
Shared decision-making is important before choosing sublobar resection or lobectomy for clinical stage I lung cancer. The surgeon should work with patient on current evidence-based information, the surgeon’s experience, and the patient’s preferences.	100.00%	0.00%	Yes
Segmentation approaches			
Segmentectomy can be proceeded from hilum or interlobar fissures considering location of segments and degree of fissure development. For more complex anatomical basal segmentectomy, one can proceed through the inferior pulmonary ligament approach and use the method of stem-branch to track the anatomy.	72.73%	18.18%	Yes
Complex segmentectomy may require more endoscopic stapler firings compared with lobectomy due to the need for longer transection lines over intersegmental planes.	90.91%	0.00%	Yes
Lymph node assessment			
The significance of lymph node dissection in patients with stage 1 NSCLC of solid-tumor undergoing segmentectomy is undefined.	72.73%	18.18%	Yes

Neutral votes make up the remaining percentages for each statement. *NSCLC*, Non–small cell lung cancer.

## Supplementary Data

Video 1 A summary of the Delphi consensus study, presented by Dr Lunxu Liu. Video available at: https://www.jtcvs.org/article/S2666-2736(23)00088-8/fulltext.
