# Supplementary figures and images for: Asia expert consensus on segmentectomy in non–small cell lung cancer: A modified Delphi study
Source: JTCVS Open. 2023 Apr 7;14:483–501. doi: 10.1016/j.xjon.2023.03.013 (PMC10328970; doi:10.1016/j.xjon.2023.03.013)

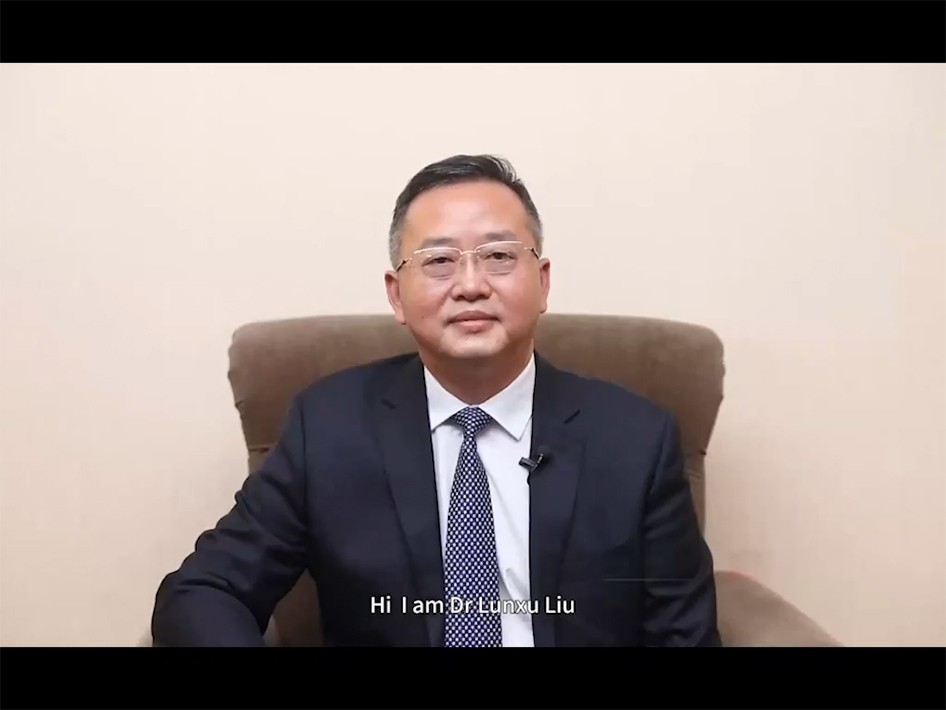

Supplement: Video 1 — A summary of the Delphi consensus study, presented by Dr Lunxu Liu. Video available at: https://www.jtcvs.org/article/S2666-2736(23)00088-8/fulltext. [file fx3.jpg]
